# 5335 days of *Implementation Science*: using natural language processing to examine publication trends and topics

**DOI:** 10.1186/s13012-021-01120-4

**Published:** 2021-04-26

**Authors:** Jonathan P. Scaccia, Victoria C. Scott

**Affiliations:** 1 Dawn Chorus Group, 1014 Hartman Road, Reading, PA 19606 USA; 2 Department of Psychological Science, University of North Carolina at Charlotte, 9201 University City Boulevard, Charlotte, NC 28223 USA

**Keywords:** Implementation science, Natural language processing, Synthesis and translation, Bibliometric study, Systematic review

## Abstract

**Introduction:**

Moving evidence-based practices into the hands of practitioners requires the synthesis and translation of research literature. However, the growing pace of scientific publications across disciplines makes it increasingly difficult to stay abreast of research literature. Natural language processing (NLP) methods are emerging as a valuable strategy for conducting content analyses of academic literature. We sought to apply NLP to identify publication trends in the journal *Implementation Science*, including key topic clusters and the distribution of topics over time. A parallel study objective was to demonstrate how NLP can be used in research synthesis.

**Methods:**

We examined 1711 *Implementation Science* abstracts published from February 22, 2006, to October 1, 2020. We retrieved the study data using PubMed’s Application Programming Interface (API) to assemble a database. Following standard preprocessing steps, we use topic modeling with Latent Dirichlet allocation (LDA) to cluster the abstracts following a minimization algorithm.

**Results:**

We examined 30 topics and computed topic model statistics of quality. Analyses revealed that published articles largely reflect (i) characteristics of research, or (ii) domains of practice. Emergent topic clusters encompassed key terms both salient and common to implementation science. HIV and stroke represent the most commonly published clinical areas. Systematic reviews have grown in topic prominence and coherence, whereas articles pertaining to knowledge translation (KT) have dropped in prominence since 2013. Articles on HIV and implementation effectiveness have increased in topic exclusivity over time.

**Discussion:**

We demonstrated how NLP can be used as a synthesis and translation method to identify trends and topics across a large number of (over 1700) articles. With applicability to a variety of research domains, NLP is a promising approach to accelerate the dissemination and uptake of research literature. For future research in implementation science, we encourage the inclusion of more equity-focused studies to expand the impact of implementation science on disadvantaged communities.

## Introduction

The classic implementations science axiom says that it takes an average of 17 years for evidence-based practices (EBPs) to be routinized in real-world settings [1–3]. This statistic has become so commonplace that it is almost an implementation science shibboleth. However, the underlying truth is that good ideas do not always get into practice. Making the first leap from research to community-based trials and then the bigger jump into routine use is laborious and complex. Addressing this challenge is the central purpose of the field [4].

The Interactive Systems Framework for Dissemination and Implementation was developed as a model to bridge the gap between research and practice [5]. It delineates three critical systems that make this happen: (i) the delivery system, comprised of individuals and settings involved in the implementation of evidence-based practices (EBPs), (e.g., healthcare clinics, school, rehabilitation centers); (ii) the support system, which builds the readiness of the delivery system for EBP implementation through training, technical assistance, and other capacity-building strategies [6]; and (iii) the synthesis and translation system, which organizes, summarizes, and translates research findings into practitioner-friendly formats. This article hones in on synthesis and translation activities through examining trends in the publication of implementation research.

The accumulation of academic literature has grown exponentially across disciplines in the last 20 years [7], including within the field of implementation science. Since the inception of *Implementation Science—*the flagship journal for peer-reviewed research on the scientific study of methods to promote the uptake of research findings into routine healthcare—the number of yearly submissions has increased eightfold, from 100 submissions in 2006 [8] to over 800 in 2018 [9]. In response to the rise and diverse nature of submissions pertaining to implementation research, a companion journal titled *Implementation Science Communication* was launched in 2020, which accepts a broader range of methodological studies. Two other implementation-specific journals were established in 2020 (*Implementation Research and Practice*; *Global Implementation Research and Action*), signifying the expanding appetite for implementation research. This does not include the multitude of journals that publish implementation science-adjacent literature (*The American Journal of Community Psychology, Health Promotion and Practice, Frontiers in Public Health*, etc).

With the rapid growth in publications, an emerging challenge to bridging the research-practice gap is the time required to synthesize research studies. The average time to complete and publish a systematic review is 67.3 weeks (1.3 years) [10]. By the time a review is published, the study data are approximately a year dated. The average time to complete a scoping review is 5.2 months, with some efforts requiring as long as 20 months [11]. The time-consuming and labor-intensive nature of systematic and scoping reviews partially account for persistent lags in moving research to practice. A fundamental challenge to synthesis and translation activities is how to extract information from research literature more effectively and efficiently [12, 13].

To accelerate the synthesis of research literature, scientists have increasingly turned to the use of natural language processing (NLP). NLP is a set of methods and computer-aided algorithms designed to detect patterns in textual data. By treating words and clustering of words as meaningful, NLP extracts concepts and relationships from texts more efficiently than humans are capable of doing. NLP is emerging as a valuable strategy for conducting content analyses of academic literature through bibliometric and scientometric studies. These studies involve the examination of digital data objects (e.g., author, journal, publisher, citations, co-wording of abstracts) to quantify study characteristics within a publication dataset [7, 12]. They can inform the impact of a search tool (e.g., Web of Science [7]), or shed light on trends within a specific research field [12, 14]. NLP has been used across a variety of disciplines and consumer applications to understand scholarly publication trends [14–17], but has not yet filtered into the methodological mainstream in social services research.

The substantial growth of implementation science, and a personal affinity for this field, sparked our interest in how to leverage these advances to examine how the field has evolved over time. We utilize an NLP approach to identify publication trends in the flagship journal in the field, *Implementation Science*, including the distribution of content areas and association among concepts. Specifically, this study aims to answer the following research questions:

1. What is the composition of content areas published in *Implementation Science*?
2. How have these content areas changed over time?

While answering these questions, we also have a methodological aim: to demonstrate how NLP can be used in research syntheses.

## Methods

### Sample

This study includes the abstracts and associated meta-data for *Implementation Science* articles published between February 22, 2006, (*Welcome to Implementation Science* [18]) and October 1, 2020. We retrieved the study data using PubMed’s Application Programming Interface (API) to assemble a database that included the following publication attributes: title, authors, affiliation, abstract, keywords, and date indexed. Of primary interest to us for this study was the publication abstracts and the data indexed. There were no applicable EQUATOR standards since we limited our database to articles published in one journal and we used all data that was indexed.

As of October 1, 2020, there were 1711 unique article abstracts available in PubMed, which we used in this analysis. We note that there appears to be a day or two lag time between when articles are uploaded to the *Implementation Science* website and when they appear in PubMed. Additionally, articles published in 2008 and 2009 were not indexed until 2010 for unknown reasons. We chose to preserve the PubMed indexing date because that is when a consumer of research would have come across the studies in PubMed.

#### Data analysis

We used several NLP methods to review the content areas in the abstracts. In this article, we treat “content areas” as the conceptual groups of constructs that are presented in the text. The core of NLP is tokenization, which treats the words as “tokens” that are meaningful information units in and of themselves. Generally, the more frequently that a token occurs, the more relevant it is in descriptive analysis. This is known as the *Bag of Words* approach. However, a fair amount of preprocessing is needed to prepare the text for analysis. We first removed *stop words*, or words that add little informative value to the overall text, such as “and,” “the,” and “with.” We then *lemmatized* the remaining words. Lemmatization involves converting a word down to its root form [19]. In some cases, this involves making plural words singular. It also involves normalizing the tense of the word (which is especially relevant when dealing with irregular verbs in English, such as “be”). A similar, less intensive method, *stemming*, is sometimes used because it is less computationally expensive than lemmatization. However, our dataset was not large enough for this to be an issue.

#### What is the composition of content areas published?

To examine study question 1, we used a topic modeling approach using Latent Dirichlet allocation (LDA). The use of “topic” here has a technical definition. LDA assumes that each *topic* is a cluster of words that co-occur together, and that documents (in this case: abstracts) are clusters of topics [20, 21]. Topics are used to represent abstract content areas. In order to identify the ideal number of topics (*k*), researchers want to find the lowest perplexity score across a number of possible topics. *Perplexity* is a metric that looks at how well a probability distribution predicts a sample. To arrive at this minimum, we tested potential topic clusters from two up to 60. While our goal was to minimize the perplexity score, we also wanted to maintain some topic interpretability. This can be a tradeoff; while we may show mathematical improvements, several of the emergent topics may strain a clear interpretation. We decided on a parameter of *k* = 30 topics. While we were continuing to show improvements in the perplexity at higher *k*s, too many of the emergent topics did not appear to add conceptual value. This is akin to finding the “bend in the curve” in principal components analysis when we begin to see diminishing returns in improvement in the metric of choice. Topic modeling is a Bayesian process [21], so to ensure replicability of our analysis, we set a seed number.

An LDA yields a matrix where each document (in this case: an abstract) is assigned a likelihood of belonging to a specific topic: the *γ* (“gamma”) value [22]. Researchers generally take the highest *γ* value to assign a document to a topic, with the caveat that this statistic is a likelihood, rather than a classification.

Topic modeling is a primarily data-driven process, though there are methods to seed the model with specific words [23]. Because of this, the LDA outputs may either not be interpretable or so broad that there is little meaning that a human could sense. Boyd-Graber and colleagues [21] developed a number of metrics to judge the quality of topics generated by an LDA approach (listed below). Like most metrics, there is no rule of thumb that guides absolute cutoff scores.

- *Topic size*: Total number of tokens by topic. There is a strong relationship between topic size and topic quality because common topics are generally represented in many documents, and are not as susceptible to being “diluted” by smaller topics [21].
- *Mean token length*: The average number of characters for the top tokens in a topic.
- *Prominence*: How many unique documents in which a topic appears. In this type of analysis, we generally find that the methodology-specific topics have higher prominence because they are more likely to feature in many articles.
- *Coherence:* How often the top tokens in each topic appears together in the same document.
- *Exclusivity*: How unique the top tokens in each topic are when compared to the other topics.

#### How have these content areas changed over time?

We assigned each article to a topic based on its maximum *γ* value, and then plotted the number of articles in that topic over time against the years indexed in PubMed. To decide which articles to visualize, we used the above metrics to filter the results so we did not end up with a time-series plot with 30 lines, all along a grayscale.

All statistics were computed in R 4.0.2 using a number of open-source packages (tidytext, topicmodels, quanteda). We used what are known as “out-of-the-box” algorithms, meaning we did not need to make any substantial changes to the underlying mathematics. All data that we used in this analysis are available, either directly as a database from the authors, through PubMed, or on *Implementation Science*’s website.

## Results

### Overall publication trends

**Fig. 1:**
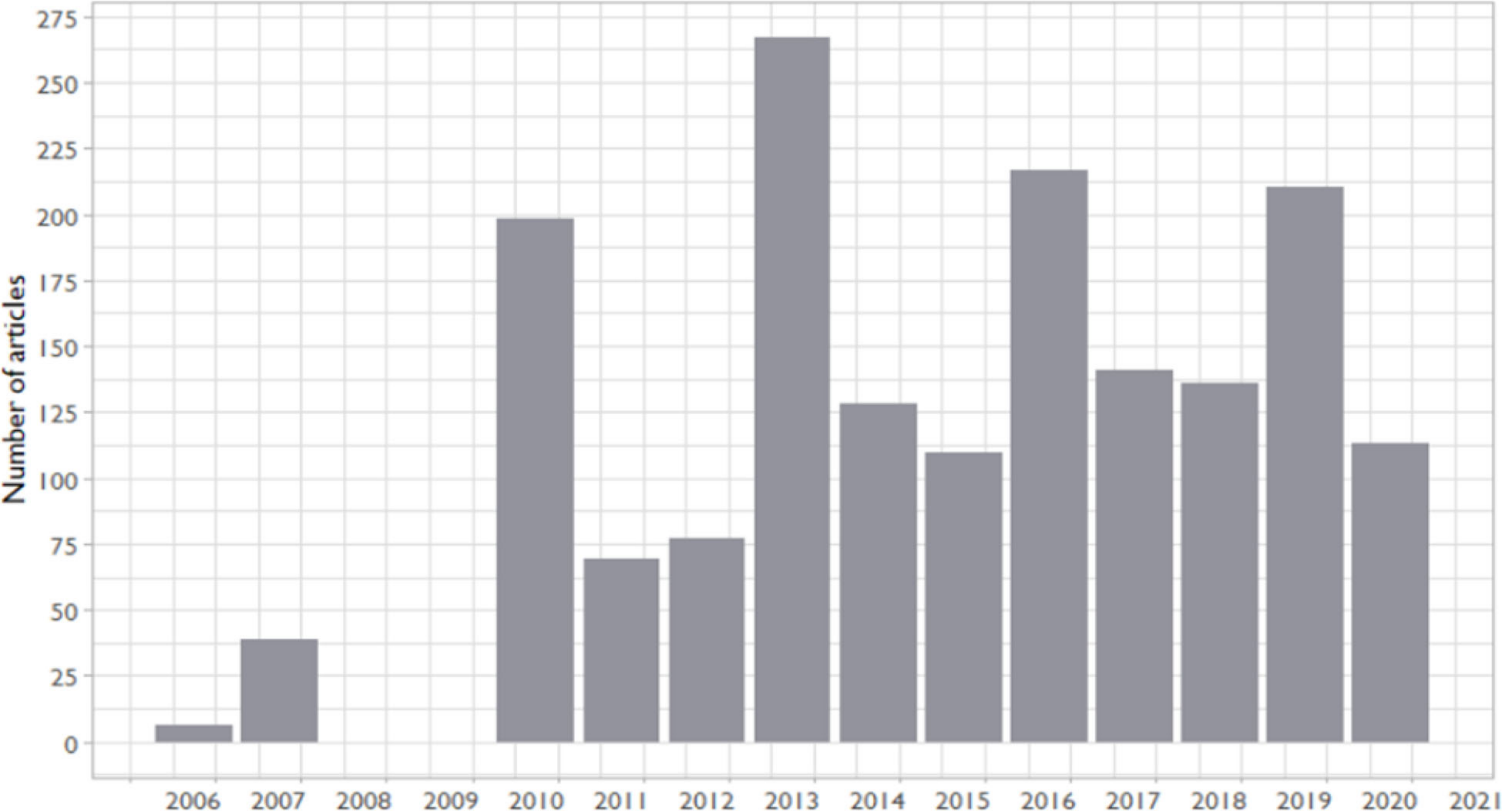
Number of published articles indexed in PubMed between 2006 and 2020

We first present general publication trend descriptive data. Apart from the PubMed indexing error for 2008-2009, analyses indicate a growth in the early years of *Implementation Science*, along with a steady volume of articles since 2014 (see Fig. 1).

**Fig. 2:**
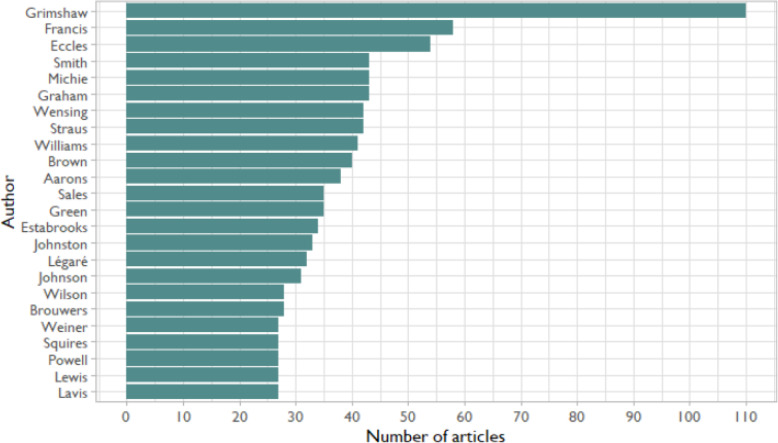
Top 25 published authors in *Implementation Science* between 2006 and 2020

We examined the 25 most frequently published authors in *Implementation Science* (Fig. 2). The total number of publications per author in this top 25 set ranged from 27-110. We made no distinction in authorship order because different fields (and even different research groups) use different conventions about the meaning of different positions (lead vs. junior researchers).

**Fig. 3:**
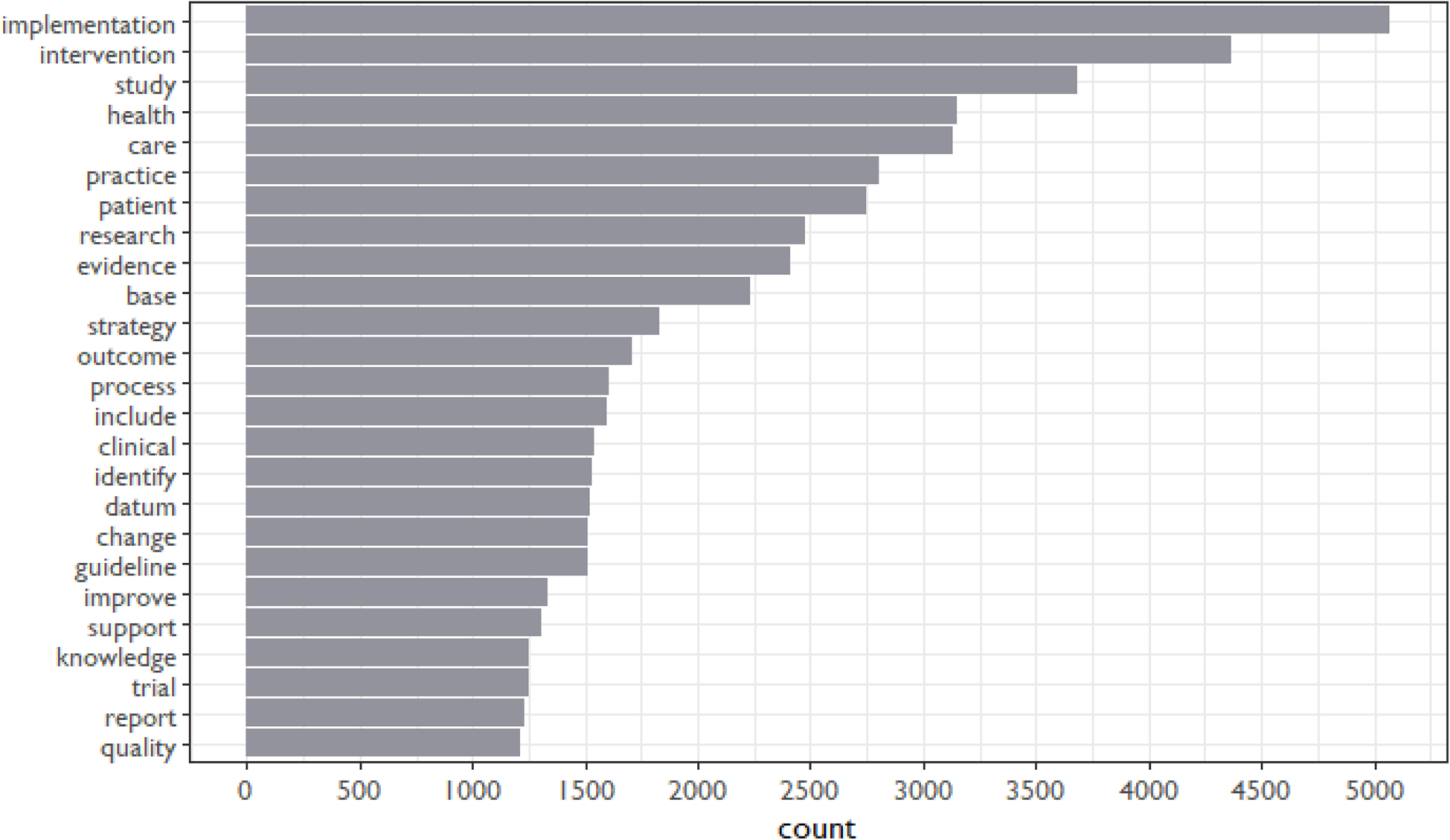
Most frequently occurring terms in *Implementation Science* abstracts

We then examined the top 25 most frequently occurring terms across all abstracts (see Fig. 3). Consistent with the focus of *Implementation Science*, we found that terms relating to health and healthcare frequently appeared, with “implementation” and “intervention” as most prevalent.

### Research question 1: What is the composition of content areas published abstracts?

**Fig. 4:**
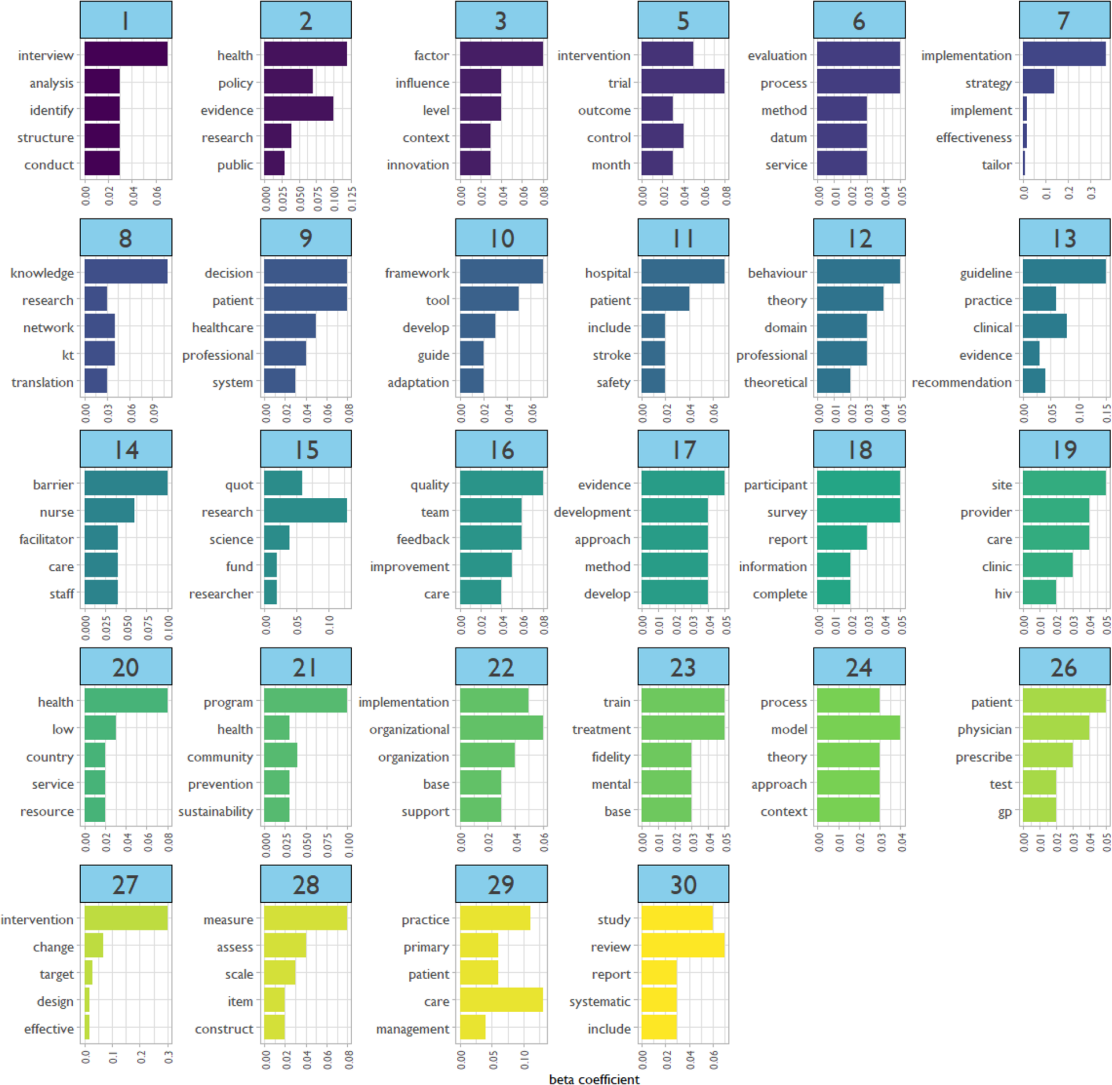
Top 5 word associations across implementation science abstract topic clusters. Note: Topic 4 and topic 25 excluded from figure due to presence of non-meaningful attributes (e.g., Ã¢, nÃ¢, 95)

We examined the composition of content areas published in article abstracts through the association of words within topics. Figure 4 depicts the top five words associated with each topic. We used five terms to avoid crowding the figure and aid data visualization. The *β* (“beta”) coefficient indicates the likelihood that a word would appear in a particular topic across all the abstracts in our data [22]. First, we used an inductive process to examine the composition of content areas published based on word associations in each topic.

We observe that the topics reflect qualities of research (e.g., topic 5: trial, outcome, control) and domains of practice (e.g., topic 28: measure, assess, construct, scale, item; topic 29: practice, primary, patient, care, management; topic 26: patient, physician, prescribe, gp, test). Two clinical health topics emerge as highly published topics: HIV (topic 19), stroke (topic 11). Prevalent settings for research are community (topic 21) and healthcare (topic 11, topic 19, topic 29.) Of note, we eliminated topics 4 and 25 from this analysis because these topic clusters appeared to collect terms that had no significant meaning. For example, the key terms of topic 4 included *Ã¢*, *nÃ¢*, *95*, *increase*, and *lt*. This type of clustering is not uncommon in high-k LDA models.

**Fig. 5:**
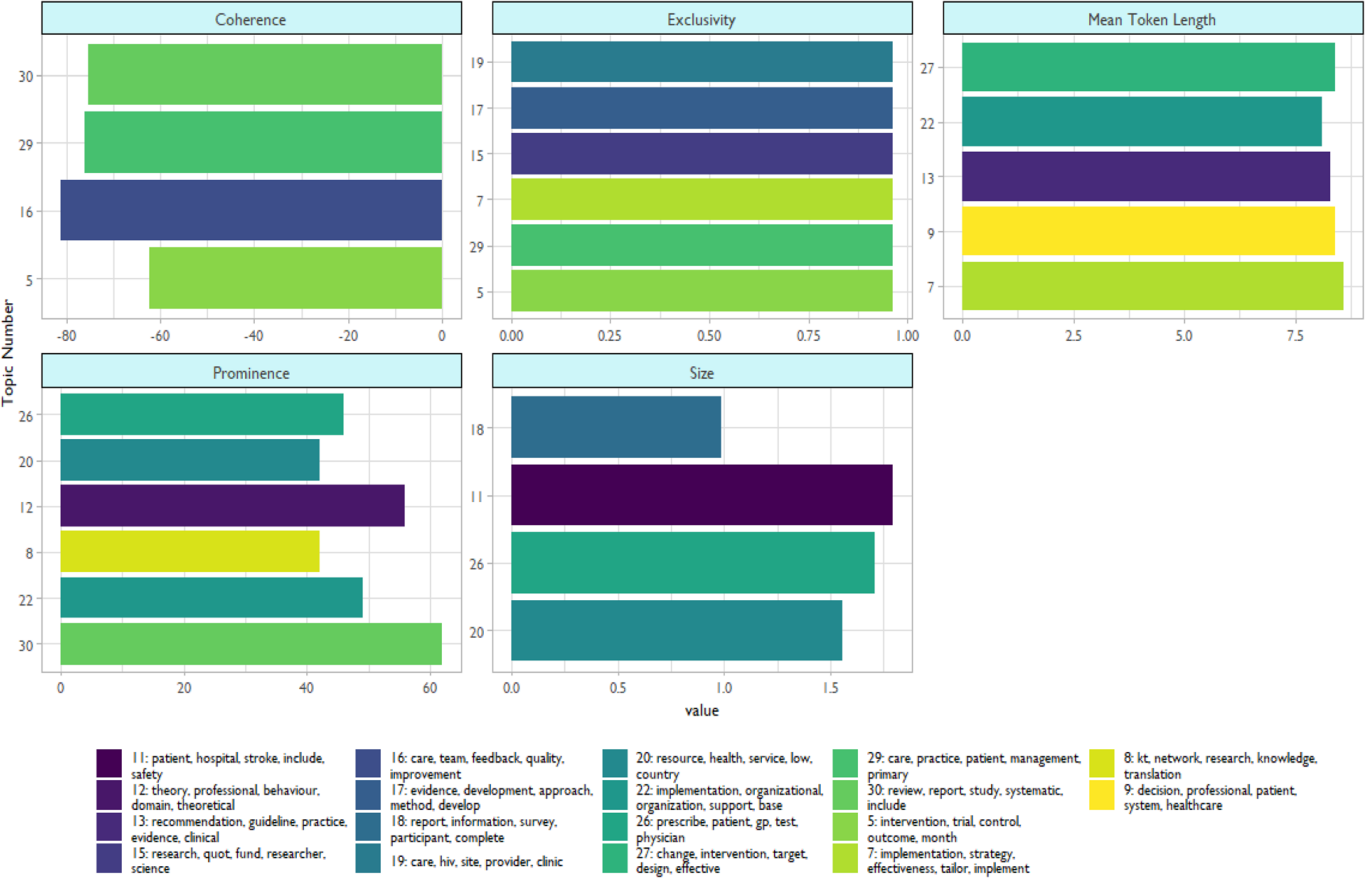
Topic clusters across the five Latent Dirichlet allocation (LDA) statistics. Note: Because the range of topic exclusivity and topic size was so narrow (as seen in Table 1), we scaled those values to better demonstrate the variations. Even so, we see a tight spread in values

While we can intuitively identify concepts across the topics (e.g., “quality improvement” in topic 16, “clinical guidelines” in topic 13, and “evidence” in topic 17), there needs to be a better method to determine the overall quality of the topic apart from a prima facie glance. Table 1 lists each topic’s empirically derived keywords by *β* coefficient, along with five LDA core statistics. Of note, we used the full model including all words to assess topic quality, not just the top five words as in Figure 4. *Topic size* refers to the number of tokens, or meaningful units of information, per topic. The largest topic sizes pertain to patient safety (potentially around stroke, topic #11). The topic with the *longest average token length*, that is the longest words, was #7 (about implementation effectiveness). Topic *prominence* is the number of abstracts in which a topic appears. The most prominent topics were #30 (pertaining to systematic reviews) and #12 (regarding behavioral domains). Topic *coherence* measures how the top tokens in each topic appear together in the same document. Values closer to zero indicated greater coherence. The most coherent topics were ##5 (pertaining to RCTs) and #30 (systematic reviews). Topic *exclusivity* is how unique the tokens in the topic are compared to other topics. Even after we scaled the data, we found the top exclusive topics had identical values. These were topics 5, 7, 15, 17, 19, and 29. Figure 5 shows the top five topics for each LDA statistics. Because we picked the topic five, there is, by definition, less variation in the scores than if we displayed all topics by these metrics.

**Table 1:** Topic model statistics

Topic	Keywords	Topic size	Mean token length	Prominence	Coherence	Exclusivity
1	Identify, analysis, interview, structure, conduct	376.84	7	16	−87.48	9.92
2	Research, policy, health, public, evidence	322.13	6.5	34	−88.49	9.94
3	Innovation, context, factor, level, influence	305.88	7.8	18	−87.81	9.94
5	Intervention, trial, control, outcome, month	298.51	7.6	33	−62.29	9.95
6	Datum, service, method, evaluation, process	328.83	7.5	19	−85.92	9.9
7	Implementation, strategy, effectiveness, tailor, implement	287.95	8.6	11	−132.68	9.95
8	KT, network, research, knowledge, translation	362.37	7.5	42	−99.38	9.87
9	Decision, professional, patient, system, healthcare	374.13	8.4	25	−94.91	9.91
10	Adaptation, tool, framework, guide, develop	326.06	6.3	22	−107.14	9.93
11	Patient, hospital, stroke, include, safety	456.9	5.9	37	−101.35	9.82
12	Theory, professional, behaviour, domain, theoretical	356.79	7.7	56	−91.61	9.92
13	Recommendation, guideline, practice, evidence, clinical	381.98	8.3	39	−107.01	9.9
14	Care, nurse, barrier, facilitator, staff	374.16	6	27	−99.46	9.91
15	Research, quot, fund, researcher, science	372.39	6.6	32	−131.6	9.95
16	Care, team, feedback, quality, improvement	317.84	6.8	31	−81.22	9.93
17	Evidence, development, approach, method, develop	323.87	8	14	−91.72	9.95
18	Report, information, survey, participant, complete	414.36	7.2	13	−103.17	9.9
19	Care, hiv, site, provider, clinic	397.64	5.6	35	−120.15	9.95
20	Resource, health, service, low, country	444.38	5.6	42	−120.73	9.9
21	Health, sustainability, program, community, prevention	368.73	7.9	24	−107.15	9.91
22	Implementation, organizational, organization, support, base	336.46	8.1	49	−103.89	9.9
23	Train, fidelity, treatment, base, mental	383.56	7.2	35	−91.36	9.88
24	Theory, model, approach, context, process	374.4	7.1	30	−89.96	9.89
26	Prescribe, patient, gp, test, physician	452.39	6.9	46	−120.72	9.9
27	Change, intervention, target, design, effective	307.89	8.4	14	−96.92	9.94
28	Item, scale, measure, construct, assess	407.97	7.4	40	−110.9	9.94
29	Care, practice, patient, management, primary	294.12	6.9	32	−76.22	9.95
30	Review, report, study, systematic, include	323.62	7.2	62	−75.42	9.93

Notes: *Topic size*, the total number of tokens by topic; higher is indicative of high quality; *Mean token length*, the average number of characters for the top tokens in a topic, with longer words potentially indicative of better quality of topics; *Prominence*, the number of unique abstracts in which a topic appears; *Coherence*, how often each topic’s top tokens appear together in the same abstract; *Exclusivity*, how unique the top tokens in each topic are when compared to the token in other topics

### Research question 2: How have these content areas changed over time?

**Fig. 6:**
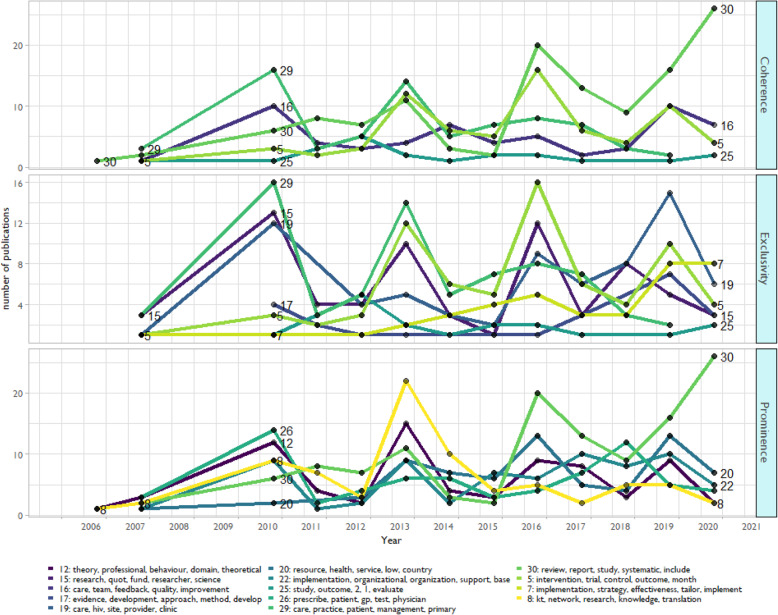
Changes in topics over time. This figure shows trends according to three metrics: (1) Prominence: how many unique abstracts in which a topic appears, (2) coherence: how often the topic tokens in each topic appears together in the same abstract, and (3) exclusivity: how unique the top tokens in each topic are compared to the other topics. The spike in 2010 is a statistical byproduct of the 2008-2009 PubMed indexing error and is not practically meaningful

Because a linear plot with 30 topic clusters is not visually pragmatic, we focused on the top five topics as ranked by *prominence*, *coherence*, *and exclusivity* (see Figure 6). The *Y*-axis is the total number of articles published overall, not a percentage representation. The spikes in 2010 are a byproduct of the PubMed 2008-2009 indexing error and are excluded from interpretation. The most notable trend is in topic 30 (systematic reviews). We see topic 30 rising in prominence and coherence. This indicates that a growth in the appearance of systematic reviews over time and that the terms in this topic cluster tightly.

We observed a drop-off in coherence for topic #5 (RCTs) since 2016. This indicates there are fewer abstracts that have the set of tokens representing these particular topics. We also see a very large drop in the prominence of knowledge translation articles (#8) after 2013, meaning the KT article appears in fewer unique abstracts overall. For exclusivity, there is a drop in primary care practice management (#29) since 2013, and a rise in HIV-specific (#19) and implementation effectiveness (#7) articles.

## Discussion

We intended this article to serve two purposes: (i) to highlight publication trends in *Implementation Science* across the tenure of the journal, and (ii) to demonstrate how NLP can be used as a method of synthesis and translation to support analyses of a large volume of publication content. Below we discuss the implications for both of these aims.

### Implications for implementation science

Implementation science is a growing field of study. Consistent with publication trends reported by Sales et al. [9], we found a positive trend in the number of articles published in *Implementation Science* between 2006 and 2020. Analyses of 28 key topics (per research question 1) revealed that published articles largely reflect characteristics of research or domains of practice. Topic clusters encompass key terms that are relevant and common to implementation science (e.g., research synthesis, decision-support, implementation effectiveness, quality improvement). HIV and stroke represent the most commonly published clinical areas.

In examining trends over time (research question 2), we found that systematic reviews have grown in topic prominence and coherence in the journal. The prominence of systematic review is consistent with trends reported by Boyd-Graber and colleagues [20], indicating that systematic reviews continue to be a unique and popular approach to research synthesis. We also found a rise in HIV-related articles overtime, with a publication spike in 2019. Articles pertaining to knowledge translation (KT) have dropped in prominence since 2013. The decreasing prominence of KT as a topic area may be unique to the study sample, and not reflective of global trends in implementation science. However, critical reviews published in other journals indicate that the scientific community is shifting away from the “knowledge translation” metaphor toward conceptualizing knowledge as being collectively negotiated and constructed—an iterative and shared process among researchers, practitioners, policymakers, and private interests [24, 25].

In reflecting on the observed trends, we asked: Could these trends relate to specific editorial changes? We believe it is possible. The editor-in-chief position turned over in 2012 (B. Mittman → A. Sales and M. Wensig) and 2019 (A. Sales → P. Wilson, with M. Wensig continuing as co-editor-in-chief). We see changes in the LDA core metrics of prominence, exclusivity, and coherence in 2013 and 2020, years which follow changes in editorial leadership. We do not have information about how much influence the editors-in-chief have over types of articles published but recognize that it is customary for journal editors writ large to encourage topic-focused publications akin to special issues. Observed trends may also be shaped by highly published authors (e.g., Grimshaw, Francis, Eccles; Table 1). For example, this could be the case if prolific authors published on a similar set of topics. While beyond the scope of our study, this is a useful association to explore for additional insight about trends in *Implementation Science* publications.

One health-related term of emerging importance that did not appear within any topic clusters was *health equity*. Broadly, health equity refers to the opportunity for all people to experience optimal health [26]. It is probable that a health equity focus is embedded within the set of examined implementation science articles, without health equity serving as a primary research outcome (e.g., study by Mizen et al. [27] and Woodward et al. [28]). However, as community psychologists, we strongly believe that quality implementation of evidence-based practices is a precursor to ameliorating underlying disparities in communities. More equity-specific research which includes the development of equitable methodologies and interventions would meaningfully expand the reach (more people have access) and bottom-line impact (it achieves better and more sustainable outcomes) of implementation science. After all, this is the primary goal: to ensure the successful uptake of evidence-based interventions in real-world settings.

### Implications for synthesis and translation

Full-time, academic researchers can find it arduous to keep pace with the scientific literature given the precipitous rise in academic publications in the recent decades [7]. The challenge of consuming research literature is greater among working practitioners involved in intervention delivery and/or the provision of care [29–31]. We have demonstrated how two specific NLP algorithms (bag of words and LDA) can be used to help identify trends and topics across a large number of (over 1700) articles. We observe that several of the topics that emerged in this analysis are evidence that NLP can pick up on these trends because they correspond to what is likely known to *Implementation Science*s’ readers and editors.

NLP is not a panacea for a deep understanding of qualitative data. There is no substitute for deeply engaging with ideas, questioning results, or contemplative analysis of issues to inform research and practice. At this time, we do not see NLP-aided methods as replacing the need to actually engage with articles. After all, there is still a large gap between NLP and natural language understanding (NLU). However, cutting-edge research such as OpenAI’s GPT-3 is moving closer to those benchmarks by using huge amounts of text-based data (175 billion parameters) to better capture nuance in language usage and how people process information [32]. We also note the human reluctance to engage with language models due to the well-founded concern that deeply embedded cultural biases will be present in results [33]. Nevertheless, NLP can be an efficient method to provide a more refined set of guideposts than one could normally obtain from querying PubMed, Google Scholar, or PLoS One. NLP provides a more advanced search and synthesis method, which may be particularly useful when approaching a new field or staying current with research. For example, when using “implementation science” as a search term, 941 articles are returned just for 2020 (as of October 22, 2020). The most dedicated researcher would be challenged to stay currently with that, let alone the practitioner pulled between client and organizational needs.

More efficient and effective synthesis and translation methods are needed in order to reduce lag in uptake of evidence-based practices. The NLP method we described addresses a barrier at the very beginning of the research-to-practice paradigm. For this article, we looked at the journal *Implementation Science.* This method can be ported to other search terms like, “decision-support,” “barrier and facilitators,” and “COVID prevention.” A more robust and refined clustering approach can assist the dissemination of scientific findings. For instance, it can help to better organize the results of a literature search process above and beyond what is normally returned with publicly available search engines. In this way, the NLP method can reduce the time required to scour the literature and provide more articles that are more representative a consumer’s interest. Additionally, NLP methods can be applied to examine correspondence between research literature and societal goals [14], implications of particular research terms [17], along with a host of other issues bearing significance in our communities [34, 35].

### Limitations

Several limitations are important to note in the context of the descriptive findings. First, the observed trends are informed by the parameters of our analysis: the use of 30 topics, and our decision to focus on trends among the top five topics. While establishing threshold limits is a common practice for NLP methods, these thresholds can shape reported trends because there is a level of analyst discretion involved [36].

We bounded our search to just *Implementation Science* the journal, not “implementation science” as a search term. This limits the generalizability of the study findings. It would be possible to replicate this analysis with all 3353 articles returned by PubMed with implementation science as a search term or by pulling in the gray literature. Indeed, looking at this larger corpus would be an interesting next step for our research, which could include more deep semantic modeling with word vectorization models that depend on data-rich inputs [37].

Also, we clustered topics, not findings. This is a critical distinction. We used topic modeling to cluster what the articles were about, not what articles reported as outcomes. Many researchers are working on developing better extractive methods to pull findings and sources of bias out of the article and into a summarization algorithm [38].

Further, topic modeling accounts for co-occurrence of words, and not the syntax or semantics of the abstract text data. The approach presented in this study does not analyze relations among words (synonyms and similar terms). This precludes in-depth insight into how particular implementation science concepts have evolved, which are better suited for literature reviews. In this study, a temporal decline in a specific topic area (e.g., knowledge translation) might indicate that a concept is going out of favor, or simply that the term used to reference the concept has evolved. Analytic approaches involving deep semantic modeling with word vectorization are a step forward though are not without their own limitations [39].

Fundamentally, we recognize the inherent challenges in human interpretation of machine-learned models. NLP has not advanced to the point where it can replace human understanding. Because the algorithms are data-driven that can lead to poor interpretations and messy results. A pure summarization-and-synthesis solution is not available yet. We acknowledge that more advanced language models are yielding incredible results. And yet, these results are only as good as the training data, and we have much work to do ensure our processes yield accurate, actionable, and ethical results [33, 40, 41].

Lastly, we identified health equity as a specific topic important for the future of implementation science. As community psychologists, equity is an anchoring value and objective. Our reflection may bear a disciplinary bias pre-conditioned by our professional training. We encourage additional reflection and group conversation about other concepts and issues that could expand the value of implementation science.

## Conclusion

By using NLP, we have demonstrated the ebbs and flows of *Implementation Science* (the journal). Our methods have captured the rise in prominence of systematic reviews, and the clustering of health and methodological-related content. We believe that similar methods can be used in a synthesis and translation process to further the bottom-line goals of *Implementation Science*. We can leverage advances in technology to accelerate the dissemination and uptake of good ideas.

## Data Availability

Study data is available at PubMed and Implementation Science. Study materials are available at: www.dawnchorusgroup.com

## Abbreviations

API: Application programming interface
LDA: Latent Dirichlet allocation
NLP: Natural language processing
KT: Knowledge translation
HIV: Human immunodeficiency virus
EBP: Evidence based practices
NLU: Natural language understanding
PCA: Principal component analysis
